# Thwarted belongingness as a factor of lower anxiety of being infected and power adherence to recommendations in pandemic in female adolescents

**DOI:** 10.1192/j.eurpsy.2022.862

**Published:** 2022-09-01

**Authors:** V. Sadovnichaja, E. Rasskazova, A. Tkhostov

**Affiliations:** 1 Moscow State University, Clinical Psychology, Mokhovaja, Russian Federation; 2 Mental Health Research Center, Medical Psychology, Moscow, Russian Federation; 3 Moscow State University, Clinical Psychology, Moscow, Russian Federation

**Keywords:** pandemic, Thwarted belongingness, Anxiety

## Abstract

**Introduction:**

Poor adherence with recommendations during pandemic is wide-spread and increases populational risk of being infected (Smith et al., 2020, Webster et al., 2020, Freeman et al., 2020). Revealing psychological factors of low adherence in adolescents is important for interventions planning. This study tests the role of perceived social support and belongingness in COVID-related anxiety and adherence.

**Objectives:**

The aim was to reveal relationships between COVID-related anxiety, monitoring of information about pandemic, adherence to recommendations and interpersonal needs in female adolescents.

**Methods:**

183 female adolescents (13-21 years old) filled Anxiety Regarding Pandemic Scale, Information Monitoring and Adherence To COVID-related Recommendations Scales (Tkhostov, Rasskazova, 2020), Interpersonal Needs Questionnaire (Van Orden et al., 2012).

**Results:**

Female adolescents moderately (m±sd=3.32±1.40 of 1–6-point scale) worried about negative consequences of pandemic on their life and lowly worried about risk of being infected (m±sd=2.53±1.15). Their adherence to recommendations was upper medium (m±sd=3.42±1.18). Neither worries nor adherence were related to age. Perceived burdensomeness was unrelated to COVID-related anxiety and adherence while thwarted belongingness was related to lower anxiety of being infected (r=-.23, p<.01) and poorer adherence to recommendations (r=-.19, p<.05).

**Conclusions:**

In female adolescents thwarted belongingness is a risk factor of poor adherence to COVID-related recommendations because of lower anxiety of being infected. Research is supported by the Russian Foundation for Basic Research, project No. 20-04-60072.

**Disclosure:**

Research is supported by the Russian Foundation for Basic Research, project No. 20-04-60072.

